# Bipolar II compared with bipolar I disorder: baseline characteristics and treatment response to quetiapine in a pooled analysis of five placebo-controlled clinical trials of acute bipolar depression

**DOI:** 10.1186/s12991-016-0096-0

**Published:** 2016-03-11

**Authors:** Catherine Datto, William J. Pottorf, Louisa Feeley, Scott LaPorte, Charlie Liss

**Affiliations:** AstraZeneca, US Medical Affairs, 1800 Concord Pike, C2C-522, Wilmington, DE 19850 USA

**Keywords:** Bipolar II disorder, Quetiapine, Clinical trials

## Abstract

**Background:**

Bipolar I and II represent the most common and severe subtypes of bipolar disorder. Although bipolar I disorder is relatively well studied, the clinical characteristics and response to treatment of patients with bipolar II disorder are less well understood.

**Methods:**

To compare the severity and burden of illness of patients with bipolar II versus bipolar I disorder, baseline demographic, clinical, and quality of life data were examined in 1900 patients with bipolar I and 973 patients with bipolar II depression, who were enrolled in five similarly designed clinical placebo-controlled trials of quetiapine immediate-release and quetiapine extended-release. Acute (8 weeks) response to treatment was also compared by assessing rating scale scores, including Montgomery–Åsberg depression rating scale, Hamilton rating scale for anxiety, Young mania rating scale, and clinical global impression-severity scores, in the bipolar I and II populations in the same pooled database.

**Results:**

Patients with bipolar I and bipolar II depression were similar in demographics, baseline rating scale scores (depression, anxiety, mania, and quality of life), and mood episode histories. Symptom improvements in response to quetiapine were greater versus comparators (lithium, paroxetine, and placebo) at 4 and 8 weeks in both bipolar I and II patients. Patients with the bipolar II subtype initially showed slower responses to all treatments, but, by 8 weeks, attained similar symptom improvement as patients with bipolar I depression.

**Conclusions:**

Pooled analysis of five clinical trials of quetiapine demonstrated that patients with bipolar II depression have a similar burden of illness and quality of life to patients with bipolar I. Bipolar II patients consistently showed a slower response to treatments than bipolar I patients, but, after 8 weeks of treatment with quetiapine, symptom improvements were similar between bipolar I and II disorder subtypes.

## Background

Bipolar I and II are the most commonly diagnosed and the most severe subtypes of bipolar disorder [1]. There is increasing evidence that the bipolar II subtype is at least as prevalent as bipolar I disorder [2] and is associated with substantial disability, comorbidity, mortality, and impact on quality of life, as recognized in the latest *Diagnostic and Statistical Manual of Mental Disorders (DSM-5) *[1, 3–6]. However, despite its prevalence and significant morbidity, the features and the treatment of bipolar II disorder have not been studied as extensively as the bipolar I subtype.

The diagnosis of bipolar I requires the presence of at least one manic episode, with or without a history of major depressive episodes, while bipolar II disorder requires at least one hypomanic and one major depressive episode [1]. Depressive episodes typically exceed manic/hypomanic episodes in duration and frequency in both bipolar I and II subtypes, so patients therefore present most frequently to physicians with depressive symptoms [7–10].

There is little evidence that the depressive symptoms and severity of bipolar I and II disorder differ. As a result, the two subtypes can be distinguished only by a careful psychiatric history that includes elucidating the presence or history of manic or hypomanic episodes, for bipolar I and bipolar II, respectively. While mania and hypomania are the core features that define bipolar I and bipolar II disorder, depressive episodes are more frequent, enduring, and disabling over the patient’s lifetime. Additionally, the frequency and duration of depressive episodes and the chronicity of illness are typically greater in bipolar II disorder [11]. Because of the challenges in correctly diagnosing hypomania, patients with bipolar II disorder are also at elevated risk of being misdiagnosed with major depressive disorder (MDD), which shares the same depressive symptoms [1, 12]. Hypomania occurs in approximately 12 % of individuals that had an initial diagnosis of MDD [1, 13]. Misdiagnosing bipolar depression as MDD may lead to the initiation of inappropriate treatment. For example, antidepressant monotherapy in patients with bipolar depression is associated with elevated rates of mood switch to mania [14–16], while the use of antidepressants as adjunctive therapy to mood stabilizers does not appear to be associated with increased efficacy in bipolar depression [17].

Relative to bipolar I disorder, there are few studies on the efficacy and safety of pharmacological treatments in bipolar II disorder. Given the greater frequency and chronicity of depressive episodes in the bipolar II subtype than in bipolar I subtype, medications that are effective in the treatment of bipolar I depression may not be directly applicable for treating depressive episodes of bipolar II disorder. As a result, there are limited recommendations in guidelines on the treatment of bipolar II depression [18–20].

**Fig. 1:**
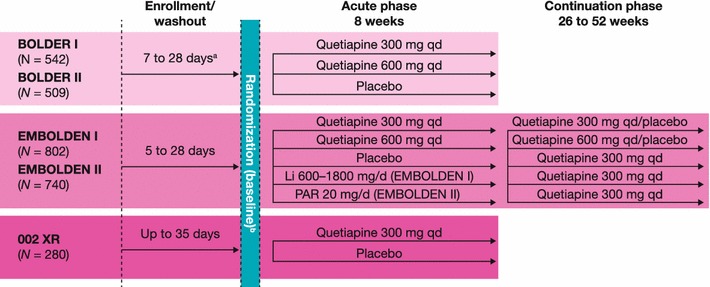
Designs of the five acute bipolar depression studies of quetiapine IR and XR. ^a^At least 5 half-lives of any prior psychotropic medications

Quetiapine monotherapy, in immediate-release (IR) and extended-release (XR) formulations, is the only US Food and Drug Administration (FDA)-approved treatment for the acute depressive episodes of both bipolar I and II disorders [20]. FDA approval in this indication was based on five placebo-controlled clinical trials that included patients with both bipolar I and II subtypes (Table 1; Fig. 1). The five trials, with similar patient inclusion and severity of illness criteria, consisted of four 8-week studies of quetiapine IR (BipOLar DEpRession [BOLDER] I and II; Efficacy of Monotherapy Seroquel in BipOLar Depression [EMBOLDEN] I and II) [21–24] and one 8-week study of quetiapine XR (Study 002 XR) [25].

**Table 1:** Overview of five acute bipolar depression studies of quetiapine IR and XR

Study name (Trial ID number/ NCT number)	Patient population	Design	Treatments	Efficacy measures
Quetiapine IR				
BOLDER I (5077US/0049/NCT00060489)^a^ BOLDER II (D1447C00135/NCT00083954)^b^	*N* = 542 (BOLDER I) *N* = 509 (BOLDER II)	8-week Double-blind, fixed-dose, parallel-group Identical study design to EMBOLDEN I and II	Quetiapine 300 mg/day or 600 mg/day Placebo	MADRS (primary) HAM-D CGI-S CGI-C PSQI (BOLDER I) SDS (BOLDER II) Q-LES-Q
EMBOLDEN I (D1447C00001)^c^ EMBOLDEN II (D1447C00134/NCT00119652)^d^	*N* = 802 (EMBOLDEN I) *N* = 740 (EMBOLDEN II)	8-week (acute phase) Double-blind, fixed-dose, parallel-group Identical study design to BOLDER I and II	Quetiapine 300 mg/day or 600 mg/day Lithium 600–1800 mg/day (EMBOLDEN I) Paroxetine 20 mg/day (EMBOLDEN II) Placebo	MADRS (primary) HAM-D CGI-BP-S CGI-BP-C HAM-A SDS Q-LES-Q (EMBOLDEN II) MOS-Cog (EMBOLDEN I)
Quetiapine XR				
002 XR (D144CC00002/NCT00422214)^e^	*N* = 280	8-week (acute phase) Double-blind, parallel-group	Quetiapine XR 300 mg/day Placebo	MADRS (primary) CGI-BP-C CGI-BP-S

*CGI*-*(BP)*-*S* clinical global impression-(bipolar)-severity, *CGI*-(*BP*)-*C* clinical global impression-bipolar-change, *HAM*-*D* Hamilton depression rating scale, *HAM*-*A* Hamilton rating scale for anxiety, *MADRS* Montgomery–Åsberg depression rating scale, *MOS*-*Cog* Medical Outcomes Study Cognitive Scale, *PSQI* Pittsburgh Sleep Quality Index, *Q*-*LES*-*Q* Quality of Life Enjoyment and Satisfaction Questionnaire, *SDS* Sheehan Disability Scale

^a^Calabrese et al. [21]

^b^Thase et al. [23]

^c^Young et al. [24]

^d^McElroy et al. [22]

^e^Suppes et al. [25]

The inclusion of both bipolar I and II patients in these quetiapine clinical trials provides an opportunity to compare the baseline demographics, illness severity, clinical characteristics, and response to treatment of patients experiencing depressive episodes of bipolar I and II subtypes.

## Methods

### Study design and patients

Retrospective pooled analyses were performed on 2873 patients who participated in five multicenter, fixed-dose, double-blind, randomized, placebo-controlled studies of quetiapine IR or XR in acute depressive episodes of bipolar I or II disorder (Table 1). Each study enrolled male or female patients aged 18–65 years with a *Diagnostic and Statistical Manual of Mental Disorders, Fourth Edition *(*DSM-IV*) diagnosis of bipolar I or II disorder, most recent episode major depression [26]. The diagnosis was confirmed by the Structured Clinical Interview for *DSM*-*IV* (SCID). Additional inclusion criteria in all studies were a Hamilton depression rating scale (HAM-D) 17-item score ≥20, HAM-D item 1 score ≥2, and Young mania rating scale (YMRS) score ≤12 [27, 28]. Hence, based on the HAM-D scale, these study populations can be considered to have depressive episodes of at least moderate severity [29]. Patients were excluded from the studies if they were diagnosed with Axis I disorder in addition to bipolar disorder.

The study designs, enrollment details, ethical approvals, and inclusion/exclusion criteria are described in detail in the original publications [21–25]. Patients in all five trials underwent a washout period of up to 28 days for antipsychotic, antidepressant, and mood-stabilizing medications prior to baseline assessments.

More details of treatment randomization procedures, dose escalation, and use of permitted co-medications are detailed in the original papers, but, in summary, quetiapine (or matched placebo) was administered orally at bedtime at a fixed dose of 300 mg/day or 600 mg/day in all studies, except in the quetiapine XR study, which included only the 300 mg/day dose. The EMBOLDEN I and II trials additionally included lithium (600–1800 mg/day) and paroxetine (20 mg/day), respectively, as active controls [22, 24]. The rationales for selecting these agents at the doses specified are provided in the original papers.

### Assessments

#### Demographic characteristics, baseline illness severity, and clinical history

Demographic and clinical assessments that were performed at baseline in all trials included: gender, age, and body weight; rating scale assessments of illness severity; and history of recent and lifetime mood episodes. Rating scale assessments performed on day 1 (after washout of previous medications) comprised the Montgomery–Åsberg Depression Rating Scale (MADRS), HAM-D, YMRS, Clinical Global Impression-Bipolar-Severity (CGI-BP-S), and Hamilton Rating Scale for anxiety (HAM-A) [30–32]. Functioning and quality of life were assessed, respectively, by two validated scales: the patient-reported Sheehan Disability Scale (SDS) and the Quality of Life Enjoyment and Satisfaction Questionnaire (Q-LES-Q) [33, 34]. Not all of these rating scales were assessed in every study, as itemized in Table 1.

#### Change in rating scale scores during quetiapine treatment

Change from baseline in MADRS total score (the primary efficacy measure) and other rating scale scores described above was assessed at study end (week 8) and at weekly or other predefined study visits. The proportions of patients who met criteria for response (i.e., MADRS score reduction ≥50 %) and remission (MADRS score ≤ 12) were also calculated in treatment groups versus placebo at study end.

### Statistical analyses

Statistical methods utilized in the individual studies are described in detail in the original papers. In brief, efficacy analyses were conducted employing a linear mixed model repeated measures (MMRM) model to analyze the difference between treatments in the change from baseline to each week. Terms were included in the model for treatments, center, and bipolar diagnosis strata as well as a term for the baseline total score as a covariate. For the current analyses of pooled data from the five clinical trials, mean and SD or SE values are provided for baseline data and response to treatment, with pooling of 300 and 600 mg dose groups for quetiapine IR in the BOLDER and EMBOLDEN studies (both quetiapine doses were significantly superior to placebo in the individual studies and in pooled analyses [35]). Efficacy of treatment was assessed in the pooled intent-to-treat (ITT) population (i.e., patients who received at least one dose of study medication and had at least one post-baseline efficacy assessment), using last observation carried forward (LOCF) methodology. No adjustments were made for multiplicity.

Analyses of safety during treatment were performed on the pooled safety population (i.e., patients who received at least one dose of the study medication). Incidences of adverse events, weight change, and changes in plasma glucose and lipid concentration during treatment are presented descriptively.

## Results

### Patients

The five clinical trials of quetiapine IR or XR investigated a total of 2873 randomized patients, including 1900 (66.1 %) with bipolar I and 973 (33.9 %) with bipolar II disorder. The proportions of patients with bipolar II disorder were similar in the BOLDER and EMBOLDEN trials (range 33–38 %) and were lower in the quetiapine XR study (19.6 %).

### Demographic characteristics, baseline illness severity, and clinical history

**Fig. 2:**
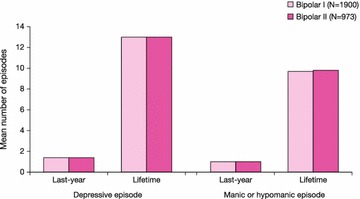
History of mood episodes in patients in bipolar I or II subgroups (pooled population)

Demographic characteristics in the pooled ITT population were broadly similar between bipolar I and II patients (Table 2). More than half the patients in both bipolar I and II subgroups were female and the mean age was approximately 39 years in both populations. Mean body weight was lower in the bipolar II than bipolar I population (79.7 vs 83.4 kg), but there was large interpatient variation. Patients with bipolar I and II disorders had similar clinical histories for the number of recent and lifetime mood episodes (Fig. 2).

**Table 2:** Demographic characteristics at baseline in bipolar I and II subgroups (ITT population)

Characteristic	Bipolar I (*n* = 1809)	Bipolar II (*n* = 922)	Total (*N* = 2731)
Gender, *n* (%)			
Male	752 (41.6)	340 (36.9)	1092 (40.0)
Female	1057 (58.4)	582 (63.1)	1639 (60.0)
Mean age (years), mean (SD)	39.6 (11.5)	38.8 (11.9)	39.3 (11.6)
Mean body weight (kg), mean (SD)	83.4 (21.4)	79.7 (19.7)	82.1 (20.9)

*ITT* intent-to-treat

**Fig. 3:**
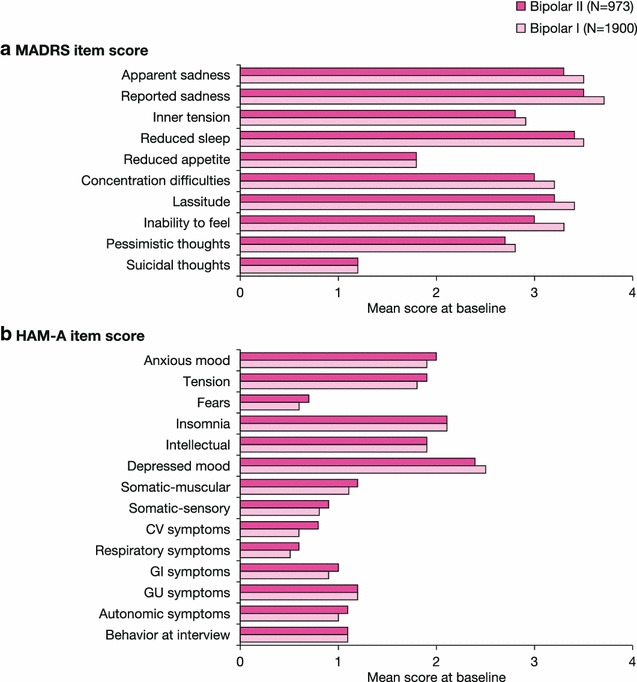
Individual item scores for depression (**a**) and anxiety (**b**) at baseline in bipolar I and II subgroups (pooled population). *HAM-A* Hamilton rating scale for anxiety, *MADRS* Montgomery–Åsberg depression rating scale. HAM-A was not measured in Suppes et al. [25]. Rating scale ranges: HAM-A: total score range 0–56; MADRS: total score range 0–60. For all rating scales, a high score represents greater severity

Mean MADRS and HAM-D scale scores at baseline were comparable between patients with bipolar I and II disorders, indicative of moderate to severe depressive symptoms in both groups (Table 3). Mean HAM-A, YMRS, and CGI-S scores were also similar between bipolar I and II patients, indicative again of similar levels of anxiety, mania/hypomania, and global rating of severity. Individual item scores on the MADRS and HAM-A scales demonstrated similar mean impairments in the core symptoms of depression and anxiety in patients with bipolar I or II disorder (Fig. 3).

**Table 3:** Symptom severity at baseline in bipolar I and II subgroups (ITT population)

Symptom rating scale	Bipolar I	Bipolar II	Total
*Depression*			
MADRS			
*n*	1809	922	2731
Mean (SD)	29.4 (6.2)	27.8 (6.7)	28.8 (6.4)
HAM-D			
*n*	1809	922	2731
Mean (SD)	24.5 (3.4)	24.1 (3.2)	24.4 (3.4)
*Anxiety*			
HAM-A^a^			
*n*	1591	868	2459
Mean (SD)	18.3 (6.3)	19.0 (6.2)	18.5 (6.3)
*Mania/hypomania*			
YMRS			
*n*	1809	922	2731
Mean (SD)	5.2 (3.1)	4.7 (2.9)	5.1 (3.1)
*Global severity of illness*			
CGI-S			
*n*	1808	922	2730
Mean (SD)	4.4 (0.7)	4.3 (0.7)	4.4 (0.7)

Rating scale ranges: CGI-S: total score range 0–7; HAM-A: total score range 0–56; HAM-D: total score range 0–52; MADRS: total score range 0–60; YMRS: total score range 0–60. For all rating scales, a high score represents greater severity

*CGI*-*S* clinical global impression-severity, *HAM*-*A* Hamilton rating scale for anxiety, *HAM*-*D* Hamilton depression rating scale, 17 item, *ITT* intent-to-treat, *MADRS* Montgomery–Åsberg depression rating scale, *YMRS* Young Mania rating scale

^a^HAM-A was not measured in Suppes et al. [25]

**Fig. 4:**
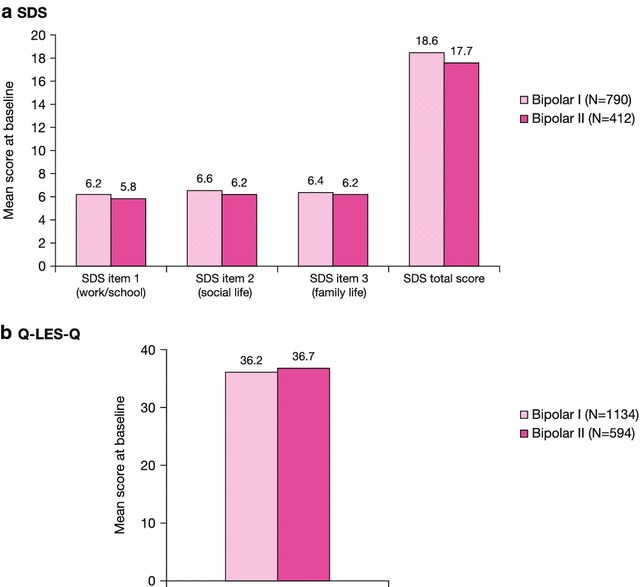
Patient-reported functioning and quality of life at baseline in bipolar I and II subgroups (pooled population). *Q-LES-Q* Quality of Life Enjoyment and Satisfaction Questionnaire, *SDS* Sheehan Disability Scale. SDS was not measured in Calabrese et al. (2005) or Suppes et al. [25]. Q-LES-Q was not measured in Young et al. [28] or Suppes et al [25]. Rating scale ranges: Q-LES-Q: total score range 14–70; a high score represents greater enjoyment/satisfaction. SDS: total score range 0–30 (individual item score ranges: 0–10); a high score represents greater severity

Patient-reported functioning (SDS total and item scores) and quality of life (Q-LES-Q total score) demonstrated severe impact in these measures at baseline and similarity between the bipolar I and II subgroups (Fig. 4). The comparability in baseline symptom severity, patient functioning, and quality of life for the bipolar I and II subtypes is in agreement with *DSM*-*5* and further highlights the severity of depressive episodes in the bipolar II subtype.

### Change in illness severity during treatment

Patients randomized to 8-week treatment in the five pooled clinical trials received quetiapine (*n* = 1162, bipolar I; *n* = 598, bipolar II), placebo (*n* = 486; 231), lithium (*n* = 87; 49), or paroxetine (*n* = 74; 44). Rates of study discontinuation in these treatment groups were quetiapine (43.1 %, bipolar I; 39.6 %, bipolar II), placebo (43.2 %; 38.7 %), lithium (42.5 %; 55.1 %), and paroxetine (63.6 %; 53.3 %, respectively).

#### Treatment group comparisons

**Fig. 5:**
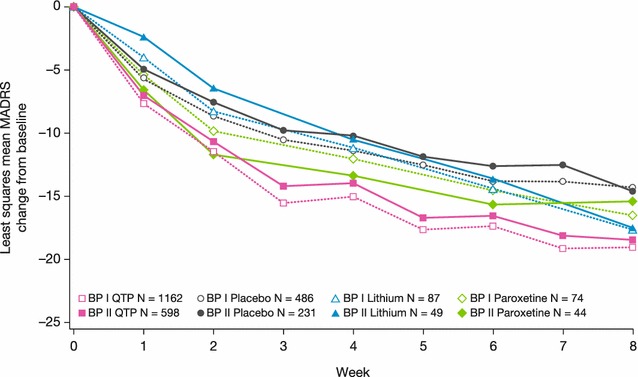
Least squares mean change from baseline to week 8 in MADRS total score (ITT population, LOCF), BPI and BPII groups. Rating scale range: MADRS: total score range 0–60. A reduction in score represents improvement in depressive symptoms

Early improvement in depressive symptoms, assessed by MADRS score change from baseline at weeks 2 and 4, was most rapid in the quetiapine group in both bipolar I and II patients, while symptom improvement was slowest in bipolar II patients treated with lithium or placebo (Fig. 5; Table 4). Least squares mean (SE) MADRS score changes at weeks 2 and 4 in bipolar I patients were −11.46 (0.24) and −15.01 (0.27) in quetiapine, −8.29 (0.87) and −11.16 (0.98) in lithium, −9.83 (0.97) and −12.01 (1.09) in paroxetine, and −8.65 (0.38) and −11.31 (0.43) in placebo groups. Changes in MADRS score at weeks 2 and 4 in patients with bipolar II were −10.66 (0.34) and −13.96 (0.38), −6.43 (1.15) and −10.46 (1.31), −11.67 (1.22) and −13.38 (1.36), and −7.52 (0.54) and −10.17 (0.60), respectively.

**Table 4:** Least squares MADRS score change (mean, SE) during treatment in bipolar I and II subgroups (ITT population)

Treatment	Bipolar I *n* = 1162			Bipolar II *n* = 598		
	n	Least squares MADRS score change (mean, SE)	Probability^a^	n	Least squares MADRS score change (mean, SE)	Probability^a^
Quetiapine						
Week 4	944	−15.01 (0.27)	<.01	484	−13.96 (0.38)	<.01
Week 8	781	−19.01 (0.30)	<.01	422	−18.44 (0.41)	<.01
Lithium		*N* = 87			*N* = 49	
Week 4	74	−11.16 (0.98)	NS	41	−10.46 (1.31)	NS
Week 8	66	−17.71 (1.03)	<.05	34	−17.46 (1.43)	NS
Paroxetine		*n* = 74			*n* = 44	
Week 4	59	−12.01 (1.09)	NS	38	−13.38 (1.36)	NS
Week 8	45	−16.54 (1.24)	NS	31	−15.35 (1.50)	NS
Placebo		*n* = 486			*n* = 231	
Week 4	388	−11.31 (0.43)	–	198	−10.17 (0.60)	–
Week 8	304	−14.29 (0.48)	–	164	−14.57 (0.65)	–

Analyses conducted using a linear mixed model (MMRM)

*ITT* intent-to-treat, *MADRS* Montgomery–Åsberg depression rating scale, *NS* nonsignificant

^a^Probability of comparison in change from baseline between treatment and placebo

During the second 4 weeks of treatment, the patient groups randomized to quetiapine continued to show symptom improvement, but the groups treated with paroxetine and placebo showed the slowest symptom improvement. The group treated with lithium nearly attained the symptom improvements achieved by the group randomized to quetiapine (Fig. 5; Table 4).

By study end at week 8, mean MADRS total score change from baseline remained greatest in the quetiapine group in both patients with bipolar I and II (Fig. 5; Table 4). In patients with bipolar I, least squares mean (SE, *p* value for comparison to placebo) MADRS score change was −19.01 (0.30, *p* < .01) in quetiapine, −17.71 (1.03, *p* < .05) in lithium, −16.54 (1.24, NS) in paroxetine, and −14.29 (0.48) in placebo groups. In patients with bipolar II, MADRS score change was −18.44 (0.41, *p* < .01), −17.46 (1.43, NS), −15.35 (1.50, NS), and −14.57 (0.65), respectively. Analyses conducted on the per-protocol population yielded very similar results (data not shown).

**Fig. 6:**
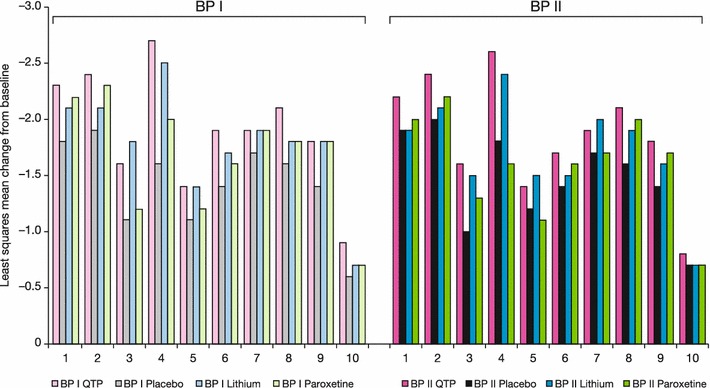
Least squares mean change from baseline to week 8 in MADRS individual item scores (ITT population, LOCF), BPI and BPII groups. Rating scale range: MADRS: all item scores range 0–6. A reduction in score represents improvement in depressive symptoms. MADRS items: (1) apparent sadness; (2) reported sadness; (3) inner tension; (4) reduced sleep; (5) reduced appetite; (6) concentration difficulties; (7) lassitude; (8) inability to feel; (9) pessimistic thoughts; (10) suicidal thoughts

With respect to individual MADRS items at week 8, the group treated with quetiapine showed the greatest improvements in most items in both bipolar I and II subgroups (Fig. 6). Items 3 (inner tension), 5 (reduced appetite), 7 (lassitude), and 9 (pessimistic thoughts) appeared to respond equally well in the lithium and quetiapine groups.

**Fig. 7:**
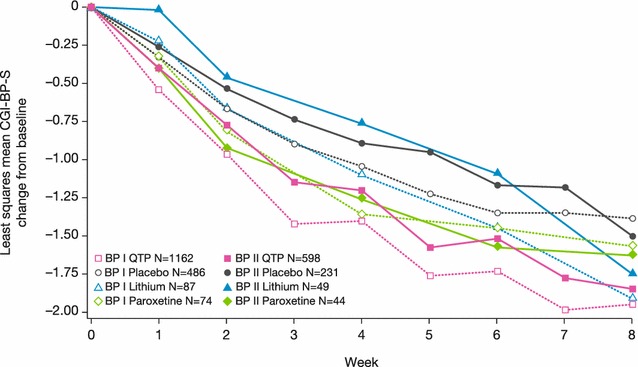
Least squares mean change from baseline to week 8 in CGI-BP-S score (ITT population, LOCF), BPI and BPII groups. Rating scale range: CGI-S: total score range 0–7. A reduction in score represents improvement in overall severity of symptoms

**Fig. 8:**
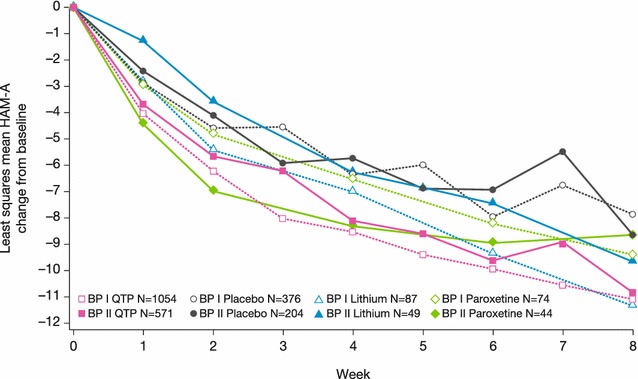
Least squares mean change from baseline to week 8 in HAM-A score (ITT population, LOCF), BPI and BPII groups. Rating scale range: HAM-A: total score range 0–56. A reduction in score represents improvement in anxiety symptoms

Similar to the changes in MADRS score, greatest improvements at week 8 in global symptom severity (assessed by CGI-S) and anxiety (HAM-A) were observed in the quetiapine and lithium groups, followed by paroxetine and placebo (Figs. 7, 8). In patients with bipolar I, least squares mean (SE) CGI-S score change was –1.95 (0.04) in quetiapine, −1.91 (0.14) in lithium, −1.57 (0.18) in paroxetine, and −1.38 (0.07) in placebo groups. In bipolar II patients, CGI-S score change was −1.84 (0.06), −1.75 (0.20), −1.62 (0.21), and −1.50 (0.09), respectively. For HAM-A score in bipolar I patients, least squares mean (SE) change was −11.11 (0.21) in quetiapine, −11.30 (0.70) in lithium, −9.43 (0.85) in paroxetine, and −7.85 (0.38) in placebo groups. In bipolar II patients, HAM-A score change was −10.84 (0.28), −9.62 (0.97), −8.65 (1.02), and −8.67 (0.47), respectively.

#### Bipolar I versus bipolar II subtype comparisons

Patients with bipolar II disorder consistently showed a slower response than bipolar I patients to all treatments, based on the week 4 assessments of mean MADRS, CGI-S, and HAM-A scores. By week 8, however, the symptom improvements in bipolar II patients approximated those in bipolar I patients for each treatment (Figs. 5, 7, 8). Comparison of MADRS items showed broadly equivalent improvements in the bipolar I and II subtypes.

### Safety assessments

An overview of adverse events and rates of the most frequent adverse events (≥5 % of the safety population) in bipolar I or II patients are reported in Tables 5 and 6. Overall adverse event rates in the treatment groups were quetiapine (76.7 %, bipolar I; 74.5 %, bipolar II), lithium (54.0 %; 65.3 %), paroxetine (71.1 %; 66.7 %), and placebo (72.4 %; 66.5 %). Incidences of adverse events leading to discontinuation were, respectively, quetiapine (9.9 %; 14.2 %), lithium (5.7 %; 10.2 %), paroxetine (11.8 %; 4.4 %), and placebo (3.8 %; 4.1 %). Incidences of adverse events and of adverse events leading to discontinuation were broadly similar across the active treatment groups (Table 5). Adverse events were mild or moderate in intensity in ≥80 % of both bipolar I and II patients in all treatment groups with the exception of patients with bipolar I treated with paroxetine (66.7 %).

**Table 5:** Overview of adverse events (safety population)

AE category^a^	Quetiapine		Placebo		Lithium		Paroxetine	
	BPI (*n* = 1217)	BPII (*n* = 632)	BPI (*n* = 500)	BPII (*n* = 242)	BPI (*n* = 87)	BPII (*n* = 49)	BPI (*n* = 76)	BPII (*n* = 45)
Any AE, *n* (%)	933 (76.7)	471 (74.5)	362 (72.4)	161 (66.5)	47 (54.0)	32 (65.3)	54 (71.1)	30 (66.7)
Any AE leading to discontinuation of treatment, *n* (%)	120 (9.9)	90 (14.2)	19 (3.8)	10 (4.1)	5 (5.7)	5 (10.2)	9 (11.8)	2 (4.4)
Any SAE (including outcome of death), *n* (%)	46 (3.8)	8 (1.3)	24 (4.8)	2 (0.8)	2 (2.3)	1 (2.0)	8 (10.5)	1 (2.2)
Any SAE leading to discontinuation of treatment, *n* (%)	30 (2.5)	4 (0.6)	13 (2.6)	1 (0.4)	2 (2.3)	0 (0.0)	5 (6.6)	0 (0.0)
Any other significant AE^b^, *n* (%)	17 (1.4)	12 (1.9)	5 (1.0)	1 (0.4)	3 (3.4)	0 (0.0)	1 (1.3)	1 (2.2)

Summary tables for AEs include those reported between the first dose and 30 days final dose of study medication

*AE* adverse event, *BP* bipolar, *SAE* serious AE

^a^Patients with multiple events in the same category are counted only once in that category

^b^Any AE that led to dose of treatment being changed or temporarily stopped, or deemed by the sponsor to be significant, excluding AEs reported as SAEs or led to discontinuation of treatment

**Table 6:** Adverse events occurring in greater than or equal to 5 % of patients in any group by preferred term in decreasing frequency (safety population)

	Quetiapine		Placebo		Lithium		Paroxetine	
	BPI (*n* = 1217)	BPII (*n* = 632)	BPI (*n* = 500)	BPII (*n* = 242)	BPI (*n* = 87)	BPII (*n* = 49)	BPI (*n* = 76)	BPII (*n* = 45)
Patients with events, *n* (%)	933 (76.7)	471 (74.5)	362 (72.4)	161 (66.5)	47 (54.0)	32 (65.3)	54 (71.1)	30 (66.7)
Dry mouth	351 (28.8)	198 (31.3)	40 (8.0)	23 (9.5)	4 (4.6)	6 (12.2)	7 (9.2)	5 (11.1)
Somnolence	287 (23.6)	130 (20.6)	28 (5.6)	18 (7.4)	5 (5.7)	7 (14.3)	1 (1.3)	6 (13.3)
Sedation	216 (17.7)	128 (20.3)	32 (6.4)	14 (5.8)	1 (1.1)	0 (0.0)	6 (7.9)	4 (8.9)
Dizziness	163 (13.4)	94 (14.9)	39 (7.8)	14 (5.8)	4 (4.6)	2 (4.1)	6 (7.9)	2 (4.4)
Headache	107 (8.8)	65 (10.3)	69 (13.8)	43 (17.8)	8 (9.2)	5 (10.2)	12 (15.8)	7 (15.6)
Constipation	103 (8.5)	49 (7.8)	16 (3.2)	11 (4.5)	2 (2.3)	2 (4.1)	6 (7.9)	0 (0.0)
Nausea	85 (7.0)	47 (7.4)	50 (10.0)	22 (9.1)	10 (11.5)	13 (26.5)	7 (9.2)	8 (17.8)
Fatigue	75 (6.2)	58 (9.2)	22 (4.4)	14 (5.8)	1 (1.1)	1 (2.0)	1 (1.3)	3 (6.7)
Dyspepsia	51 (4.2)	33 (5.2)	14 (2.8)	6 (2.5)	3 (3.4)	1 (2.0)	2 (2.6)	0 (0.0)
Upper respiratory tract infection	41 (3.4)	15 (2.4)	29 (5.8)	12 (5.0)	2 (2.3)	0 (0.0)	2 (2.6)	0 (0.0)
Diarrhea	39 (3.2)	21 (3.3)	24 (4.8)	19 (7.9)	2 (2.3)	7 (14.3)	6 (7.9)	2 (4.4)
Insomnia	34 (2.8)	10 (1.6)	33 (6.6)	15 (6.2)	8 (9.2)	4 (8.2)	8 (10.5)	8 (17.8)
Tremor	26 (2.1)	16 (2.5)	4 (0.8)	2 (0.8)	3 (3.4)	5 (10.2)	2 (2.6)	1 (2.2)
Pollakiuria	16 (1.3)	3 (0.5)	9 (1.8)	2 (0.8)	2 (2.3)	4 (8.2)	0 (0.0)	1 (2.2)
Asthenia	14 (1.2)	13 (2.1)	1 (0.2)	4 (1.7)	0 (0.0)	5 (10.2)	2 (2.6)	0 (0.0)
Decreased appetite	15 (1.2)	6 (0.9)	3 (0.6)	5 (2.1)	0 (0.0)	0 (0.0)	4 (5.3)	2 (4.4)
Influenza	15 (1.2)	17 (2.7)	9 (1.8)	6 (2.5)	2 (2.3)	1 (2.0)	0 (0.0)	3 (6.7)
Anxiety	13 (1.1)	17 (2.7)	13 (2.6)	10 (4.1)	4 (4.6)	1 (2.0)	3 (3.9)	3 (6.7)

The profiles of adverse events reported in the pooled analyses are consistent with the known adverse event profiles of these agents, and as reported and discussed in the original papers [21–25]. Dry mouth, somnolence/sedation, and dizziness were the most common adverse events above placebo rates in the quetiapine group; nausea, diarrhea, and tremor were common adverse events associated with lithium; and nausea and insomnia were common events in the paroxetine group.

There were no consistent differences in the incidence or profile of adverse events between patients with bipolar I and II disorder.

Weight change and changes in plasma glucose and lipid concentrations during treatment are reported in Table 7. Patients in the quetiapine group experienced a mean weight gain, while the metabolic parameters investigated showed no consistent differences between treatment groups or between patients with bipolar I and II disorder.

**Table 7:** Weight, plasma glucose, and lipid data (safety population)

Mean (SD) change from randomization	Quetiapine		Placebo		Lithium		Paroxetine	
	BPI (*n* = 1217)	BPII (*n* = 632)	BPI (*n* = 500)	BPII (*n* = 242)	BPI (*n* = 87)	BPII (*n* = 49)	BPI (*n* = 76)	BPII (*n* = 45)
Mean weight change (kg)	1.1 (4.8)	1.3 (5.5)	−0.0 (2.5)	0.1 (2.4)	0.1 (2.1)	0.3 (2.2)	−0.4 (3.2)	−0.0 (1.9)
Weight change ≥ 7 %, *n* (%)	85 (7.0)	44 (7.0)	11 (2.2)	5 (2.1)	2 (2.3)	1 (2.0)	2 (2.6)	1 (2.2)
Glucose (mg/dL)	4.6 (21.5)	3.0 (14.4)	4.0 (20.3)	2.4 (14.6)	2.2 (18.9)	2.8 (11.8)	3.3 (13.2)	−1.9 (15.3)
Insulin (pm/L)	49.9 (192.7)	27.9 (111.1)	15.6 (142.3)	19.1 (112.6)	6.0 (120.1)	−18.3 (101.2)	14.5 (83.9)	15.0 (100.4)
Total cholesterol (mg/dL)	−1.8 (31.9)	−0.9 (31.6)	−3.3 (27.6)	−4.9 (31.2)	−3.0 (37.9)	−9.3 (31.5)	−4.7 (37.9)	0.3 (30.9)
LDL-cholesterol (mg/dL)	−3.1 (28.2)	−2.8 (26.5)	−3.2 (24.3)	−5.1 (23.5)	−3.9 (32.6)	−6.8 (28.0)	−5.9 (32.8)	−3.3 (27.7)
HDL-cholesterol (mg/dL)	−0.9 (9.4)	−1.0 (9.3)	0.2 (8.8)	−1.3 (8.9)	−0.9 (13.3)	0.0 (8.9)	0.6 (12.4)	1.1 (10.2)
Triglycerides (mg/dL)	18.6 (112.8)	16.9 (85.1)	−1.1 (111.8)	8.7 (112.2)	2.8 (81.1)	−0.9 (62. 6)	−5.0 (106.8)	26.9 (91.5)

## Discussion and conclusions

Bipolar II disorder has historically been perceived as less severe and disabling than bipolar I disorder. More recent reports suggest that the chronicity, frequency, and duration of depressive episodes are typically greater in bipolar II than bipolar I disorder; while both disorder subtypes are equally disabling and impact quality of life outcomes. However, despite a similar prevalence and significant morbidity, the features and the treatment of bipolar II disorder have not been studied as extensively as the bipolar I subtype, providing limited evidence in the management of patients with bipolar II disorder.

This pooled analysis investigated baseline characteristics in a large (*n* = 2873) population of patients with bipolar I or II disorder enrolled in five acute treatment trials of quetiapine IR and XR in bipolar depression. In contrast to previous reports (e.g., Weinstock et al. [11]), the patients with bipolar II disorder enrolled in these studies had a generally similar burden of illness history as bipolar I disorder, while also confirming previous reports of similar baseline disability and impact on quality of life for both bipolar subtypes [1, 5, 6]. Since clinical features at the time of depressive episodes may not differentiate these two disorders or MDD, these findings suggest careful inquiry into the chronicity of depressive episodes and of episodes suspicious for mania or hypomania can be important to establish an accurate diagnosis.

Changes in the severity of depression, anxiety, and overall burden of illness during 8 weeks of treatment in the five pooled, randomized clinical trials reported in this paper indicate that quetiapine provided earlier and greater symptom improvements than lithium or paroxetine. Interestingly, patients with bipolar II typically responded more slowly to treatments than patients with bipolar I, but by 8 weeks the symptom improvements were similar in the two bipolar subgroups. Lithium treatment also showed an initial delay in symptom improvement for both bipolar subtypes, but by 8 weeks approached a similar symptom improvement to quetiapine treatment. Conversely, paroxetine treatment initially followed symptom improvement similar to quetiapine; however, by 8 weeks improvement slowed and was similar to placebo. Statistical comparisons versus placebo indicated that quetiapine alone provided significant benefit at both 4 and 8 weeks and in both bipolar I and II populations.

Descriptive assessments of the safety profile of the medications revealed no new findings relative to the original publications, with broadly similar incidences of adverse events and of adverse events leading to discontinuation for all active treatments in both bipolar I and II populations.

Limitations of these pooled analyses include their post-hoc nature, the generalizability of the outcomes in the context of the studies’ selection criteria that excluded significant comorbidities, and the acute duration of the five studies. We are, however, aware of no similar analyses in the literature that have compared baseline characteristics and treatment response in bipolar II versus bipolar I patients by a pooled analysis of clinical trial data.

The observations made here, of a potentially slower response by bipolar II patients in all treatment arms, should inform prescribers and patients on expectations for improvement and when to consider changes in treatment regimen versus longer watchful waiting.

## Abbreviations

CGI-BP-S: clinical global impression-bipolar-severity
*DSM-IV*: *Diagnostic and Statistical Manual of Mental Disorders*, Fourth Edition
FDA: Food and Drug administration
HAM-A: Hamilton rating scale for anxiety
HAM-D: Hamilton depression rating scale
IR: immediate release
ITT: intent-to-treat
LOCF: last observation carried forward
MADRS: Montgomery–Åsberg depression rating scale
MDD: major depressive disorder
Q-LES-Q: Quality of Life Enjoyment and Satisfaction Questionnaire
SCID: Structured clinical interview for *DSM*-*IV*
SDS: Sheehan Disability Scale
XR: extended release
YMRS: Young mania rating scale
